# Functionalized Positive Nanoparticles Reduce Mucin Swelling and Dispersion

**DOI:** 10.1371/journal.pone.0015434

**Published:** 2010-11-10

**Authors:** Eric Y. T. Chen, Yung-Chen Wang, Chi-Shuo Chen, Wei-Chun Chin

**Affiliations:** Bioengineering, University of California Merced, Merced, California, United States of America; Instituto de Tecnologia Química e Biológica, Portugal

## Abstract

Multi-functionalized nanoparticles (NPs) have been extensively investigated for their potential in household and commercial products, and biomedical applications. Previous reports have confirmed the cellular nanotoxicity and adverse inflammatory effects on pulmonary systems induced by NPs. However, possible health hazards resulting from mucus rheological disturbances induced by NPs are underexplored. Accumulation of viscous, poorly dispersed, and less transportable mucus leading to improper mucus rheology and dysfunctional mucociliary clearance are typically found to associate with many respiratory diseases such as asthma, cystic fibrosis (CF), and COPD (Chronic Obstructive Pulmonary Disease). Whether functionalized NPs can alter mucus rheology and its operational mechanisms have not been resolved. Herein, we report that positively charged functionalized NPs can hinder mucin gel hydration and effectively induce mucin aggregation. The positively charged NPs can significantly reduce the rate of mucin matrix swelling by a maximum of 7.5 folds. These NPs significantly increase the size of aggregated mucin by approximately 30 times within 24 hrs. EGTA chelation of indigenous mucin crosslinkers (Ca^2+^ ions) was unable to effectively disperse NP-induced aggregated mucins. Our results have demonstrated that positively charged functionalized NPs can impede mucin gel swelling by crosslinking the matrix. This report also highlights the unexpected health risk of NP-induced change in mucus rheological properties resulting in possible mucociliary transport impairment on epithelial mucosa and related health problems. In addition, our data can serve as a prospective guideline for designing nanocarriers for airway drug delivery applications.

## Introduction

A myriad of functionalized-NPs have been used to develop commercial commodities in various industrial sectors. They are heavily implemented in the manufacturing of paint, carpet, paper, plastic, textiles, clothes and cosmetics [1], [2], [3], [4]. NPs also play major roles in decontaminating aqueous wastes and food processing [4], [5], [6]. Recent developments in the pharmaceutical field focus on nanocarriers that transport drugs or genes to targeted areas via inhalation to treat numerous respiratory and systemic diseases [7], [8]. The pulmonary route proves to be an important site of nano-drug (e.g. aerosolized nanopowder) and therapeutic gene delivery that offers both local and systemic targeting for treatment of many diseases [8], [9]. Even in major respiratory diseases, such as pulmonary tuberculosis, cystic fibrosis (CF), Chronic Obstructive Pulmonary Disease (COPD) and bronchial asthma, nanocarriers have shown promising therapeutic effects *in vitro* and in animal experiments [9], [10].

Due to their diverse applications, recurrent aerosolized exposure to these NPs are anticipated, yet the potential health risks brought about by NPs are not fully understood [11]. Epidemiological studies have confirmed a positive correlation between levels of particulate pollution and increased morbidity and mortality rates among general populations [12], [13]. The adverse health effects seem to be dominated by pulmonary symptoms. For instance, many reports have addressed that occupational exposure of inhaled NPs can lead to respiratory diseases such as pneumoconiosis (pulmonary fibrosis) and bronchitis [14], [15]. Increasing inhalation of ambient ultrafine particles has been linked with exacerbation of respiratory symptoms and mortality among COPD sufferers [16], [17], [18]. It has also been documented that NPs can instigate oxidative stress and cellular toxicity in various types of cells [8], [19]. For instance, in human bronchial epithelial cells, we have previously demonstrated that titanium dioxide NP exposure resulted in cell death without photoactivation [1]. In addition, polystyrene NPs have been shown to induce lung inflammation [20].

Mucus rheology plays a critical role in maintaining respiratory health. Mucins are large, highly glycosylated proteins [21], [22]. The polyanionic nature of mucin stems primarily from sialic acid, sulfate, and carboxyl (COO^-^) groups present in these linked oligosaccharides. Beside physical entanglement, cationic calcium ions can act as crosslinkers that condense the mucin matrices inside mucin granules before exocytosis [22]. Upon release, phase transition mainly driven by the Donnan effect triggers the massive decondensation of mucin networks [22]. Hydrogen bonding, hydrophilic and hydrophobic interactions have also been proposed to contribute to the gel properties of mucin [22]. The gel characteristics and rheological properties of mucin are critical for maintaining the integrity of epithelia by trapping bacteria and viruses for mucociliary clearance.

The clinical manifestation of major respiratory diseases (i.e. COPD [23], asthma [24] and CF [25]) are related to thick mucus. The relationship between mucin dehydration and defective mucus clearance has been well established [26], [27], [28]. As a result, the poorly hydrated, highly viscous and less transportable mucus appears to accumulate within airway passages [24], [29]. Obstruction of airway lumen with viscous mucus is usually accompanied by chronic bacterial infection, inflammation and impaired mucociliary transport [23], [29]. Chronic exposure to NPs can potentially predispose humans to lung inflammation and increase the risk of COPD [18], [30]. However, whether NPs can alter the viscoelastic property of mucus and lead to concomitant pathological outcomes is not clear. Despite evidence on NP-induced cellular nanotoxicity, studies addressing the link between the surface property of NPs and their harmful effects are still lacking. Our research examined the relationship between NP surface modifications and alteration in mucus rheological properties. The concentration range of NPs used in this investigation was within the level found in ambience and in nanotechnology industries [20], [31]. In this study, we found that positively-charged NPs can promote mucin aggregation, thereby reducing the rate of mucin gel expansion via crosslinking polyanionic mucin matrices. Our results provide the first mechanistic link between NP exposure and viscous mucus accumulation commonly found in pulmonary diseases.

## Materials and Methods

### A549 cell culture

The human lung carcinoma cell line A549 was obtained from American Type Culture Collection (ATCC, VA, USA). A549 cell line is an airway epithelial cell line commonly used as a secretory model [32]. Cells were cultured in 15 cm cell culture plates (VWR, CA, USA) containing F-12 nutrient mixture medium (Invitrogen, CA, USA) that was supplemented with 100 U of penicillin/streptomycin and 10% heat-inactivated fetal bovine serum (FBS) (Invitrogen, CA, USA). The A549 lung cells were cultured in 15 cm Falcon plates and incubated in a humidified incubator at 37°C/5% CO_2_. Cell counts were performed using trypan blue (Sigma-Aldrich, MO, USA) exclusion and a Bright-Line haemocytometer.

### Nanoparticle preparation

Carboxyl-, amine- and non-functionalized polystyrene particles with various sizes (i.e. 57 nm, 99 nm, 120 nm and 160 nm) (Bangs Laboratories, Fishers, IN, USA) were used in our study. All NPs have a size standard deviation of ≤10% (based on manufacturer information). These sizes were independently confirmed using homodyne dynamics laser scattering. All nanoparticle samples were sonicated before usage.

### Particle sizing using dynamic laser scattering

The aggregation of mucus was monitored by measuring particle size using homodyne dynamics laser scattering (DLS). Samples of porcine gastric mucin at 1 mg/L (Sigma-Aldrich, MO, USA) were prepared with Hanks' solution containing 1.2 mM Ca^2+^, 20 mM Tris-HCl (tris(hydroxymethyl)aminomethane hydrochloride) and 10 mM MES (2-(N-morpholino)ethanesulfonic acid) (Sigma-Aldrich, MO, USA) to buffer the pH around 7.4 [33]. The solution was thoroughly mixed until mucin has dissolved. Aliquots of mucin samples (10 ml) were directly filtered through a 0.22-µm Millipore PES membrane (pre-washed with 0.1 N HCl) (Fisher Scientific, CA, USA) into clean scintillating vials. The scintillating vials were positioned in the goniometer of a Brookhaven laser spectrometer (Brookhaven Instruments, NY, USA). Mucin gel aggregation was allowed to take place by equilibrating with three filtered types of NPs each expressing different surface modifications for 72 hrs; they were subsequently analyzed by detecting the scattering fluctuations at a 45 degree scattering angle. Commercialized polystyrene NPs (Bangs Laboratories, Fishers, IN) with dimensions of 57, 99, 120 and 160 nm were added to mucin samples. Positive (-NH_2_ based, 57 and 160 nm), negative (-COOH, 120 nm) and non-functionalized (hydrophobic, 99 nm) surface charges were used in this study. All NPs were prepared in suspension of Hanks' solution (buffered with Tris-HCl/MES at pH 7.4). Filtered NP solution, added to scintillating vials, was tested for its ability to promote mucin aggregation and was monitored at 1 hr, 3 hrs, 5 hrs, 24 hrs, 48 hrs and 72 hrs. Self aggregation induced by NP-only and mucin-only controls were monitored at 1 hr, 24 hrs, 48 hrs and 72 hrs. The pH was also monitored and maintained at approximately 7.4 during the experiments. The autocorrelation function of the scattering intensity fluctuations was averaged over a 3-min sampling time using a Brookhaven BI 9000AT autocorrelator. Particle size distribution was calculated by CONTIN [34]. Calibration was conducted with standard monodisperse suspensions of latex microspheres ranging from 50 nm to 10 µm (Polysciences, PA, USA).

### Scanning electron microscopy (SEM)

Samples of porcine gastric mucin at 1 mg/L (Sigma-Aldrich, MO, USA) were prepared with Hanks' solution (buffered with Tris-HCl/MES at pH 7.4) and incubated with 10 mg/L of positively-charged 57 and 160 nm NPs. After incubation for 5 hrs or 72 hrs, 5 ml of mucin were filtered through a 0.22-µm Millipore isopore membrane (Fisher Scientific, CA, USA). The filtered mucin was subsequently fixed with 4% paraformaldehyde (prepared in PBS at pH 7.4) (Sigma-Aldrich, MO, USA) for 5 min, followed by careful rinsing with Hanks' solution twice. The fixed mucin and NP complexes were dehydrated by soaking in serially diluted ethanol (35%, 50%, 70%, 95% and 100% ethanol) for 5 min. The specimens were air dried and examined with FEI Quanta 200 ESEM (North America NanoPort, OR, USA).

### EGTA dispersion of aggregated mucin

EGTA (Ethylene glycol-bis(2-aminoethylether)-N,N,N',N'-tetraacetic acid, 2 mM) (Sigma-Aldrich, MO, USA) was used to disperse aggregated mucus, which was monitored by measuring particle size by homodyne dynamics laser scattering. Samples of porcine gastric mucin at 1 mg/L (Sigma-Aldrich, MO, USA) were prepared with Hanks' solution containing 1.2 mM Ca^2+^ (buffered with Tris-HCl/MES at pH 7.4) and thoroughly mixed until mucin has dissolved. Aliquots of mucin samples (10 ml) were directly filtered through a 0.22-µm Millipore PES membrane (pre-washed with 0.1 N HCl) (Fisher Scientific, CA, USA) into clean scintillating vials. The scintillating vials were positioned in the goniometer of a Brookhaven laser spectrometer (Brookhaven Instruments, NY, USA). Mucin gel aggregation was initiated by incubating mucin samples with filtered positively-charged NPs (-NH_2_ based 57 and 160 nm) (Bangs Laboratory Inc, Fishers, IN) and were monitored after 1, 3, 5, 24, 48 and 72 hrs. EGTA (2 mM) was subsequently applied to already aggregated mucin solution after 72 hrs. The change in aggregation size was detected for another 72 hrs. The pH was also monitored and maintained at approximately 7.4 during the experiments. The autocorrelation function of the scattering intensity fluctuations was averaged over a 3-min sampling time, using a Brookhaven BI 9000AT autocorrelator. Particle size distribution was calculated by the CONTIN [34]. Calibration was conducted with standard monodisperse suspensions of latex microspheres ranging from 50 nm to 10 µm (Polysciences Inc, PA, USA).

### Swelling kinetics of exocytosed mucin matrices

The A549 cell culture plates were rinsed with Hanks' buffer twice. Non-trypsin dissociation buffer (Invitrogen, CA, USA) was added to detach cells from the plates and were subsequently incubated at 37°C for 15 minutes, centrifuged at 700 rpm for 5 min, and resuspended in Hanks' solution (Invitrogen, CA, USA) (buffered with Tris-HCl/MES at pH 7.4). Cells were resuspended into MatTek glass bottom dishes (MatTek Corporation, MA, USA) and equilibrated in a 37°C incubator for 10 minutes prior to adding 10 mg/L and 1 mg/L of 160 and 57 nm positively-charged NPs (Bangs Laboratories, Fishers, IN). The pH was monitored and maintained at approximately 7.4 throughout the experiments.

A549 cells were viewed and video-recorded with phase-contrast lens using a Nikon Eclipse TE-2000-U inverted fluorescence microscope (Nikon Eclipse TE-2000U, Tokyo, Japan). Degranulation of A549 cells was induced by 1 uM ionomycin (Sigma-Aldrich, MO, USA) and was found to be a readily observable discrete quantal process. During exocytosis into extracellular Hanks' solution, released granule matrices undergo rapid swelling. Video-recordings of granular exocytosis and swelling were captured at 30 frames s^−1^.

Measurements of radii, of the released mucin matrices, as a function of time were used to verify that the swelling of the secreted material followed the characteristic features of polymer gel swelling kinetics [35], [36]. The swelling of a polymer gel follows a typical diffusive kinetics that is independent of the size, internal topology, or chemical composition of the gel [36]. For spherical gels as observed from the exocytosed mucin granule matrices of A549 cells, the radial dimension increased following a characteristic first order kinetics of the form r(t) = r_f_–(r_f_–r_i_) e^–t/τ^ (equation 1), where r_i_ and r_f_ are the initial and final radius of the secretory granule matrix, respectively, and τ is the characteristic relaxation time of the swelling process [22]. The polymer network of gels diffused into the solvent (Hanks' solution), with a diffusivity (D) (D = (r_f_)^2^/τ [cm^2^ s^−1^]) (equation 2). The diffusivity (D) of polyionic gels varied with the concentration of counterions in the swelling medium.

### Statistical Analysis

The data was presented as means±SD. Each experiment was performed independently at least three times. Statistical significance was determined using a Student's t-test analysis with p values of <0.005 (Microsoft Excel and GraphPad Prism 4.0, GraphPad Software, Inc., San Diego, CA, USA).

## Results

### Positively-charged nanoparticle induces mucin aggregation

**Figure 1:**
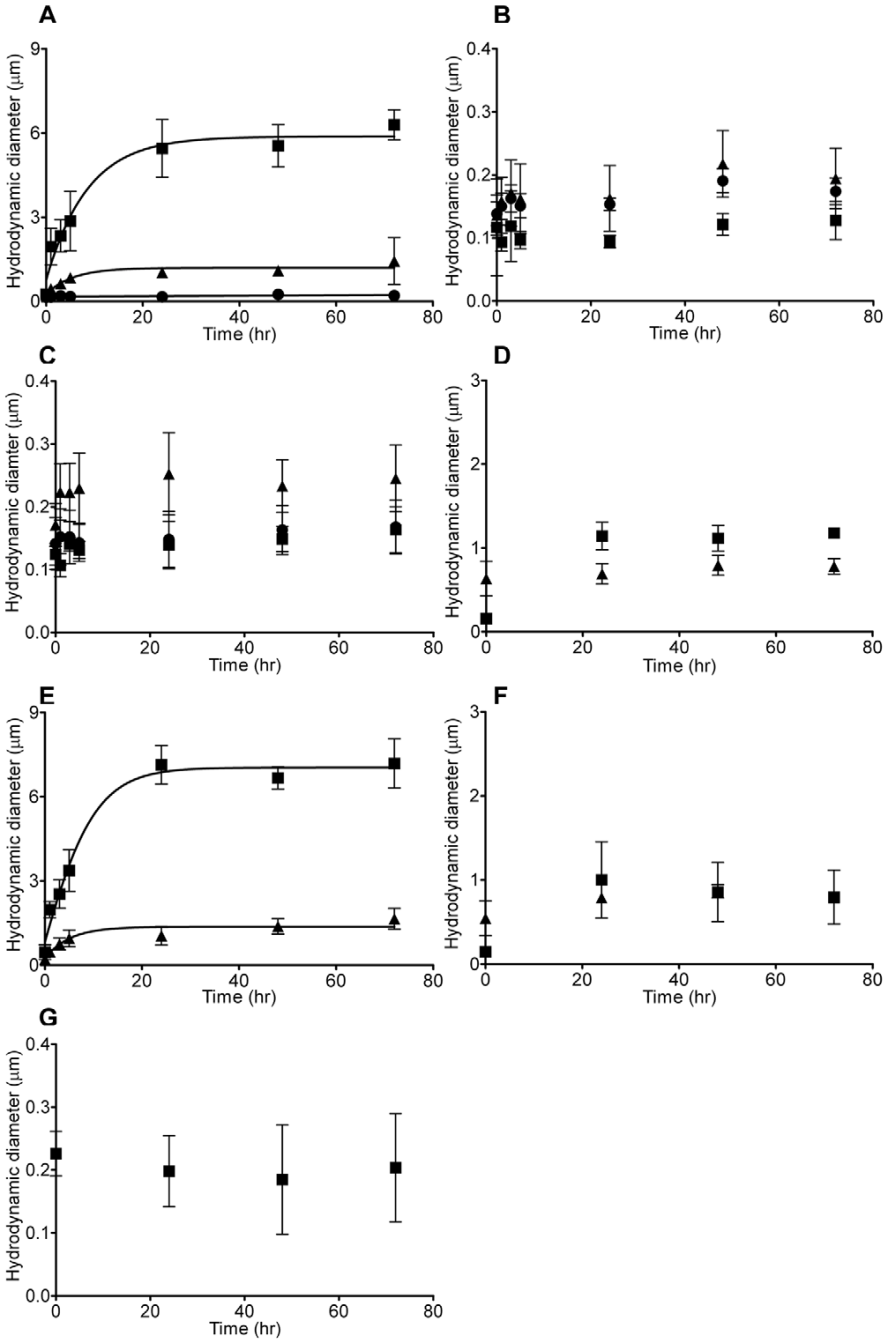
Interaction of NPs and mucin aggregation. (A) Positively-charged NPs (160 nm) (n≥6) induce significant mucin aggregation while (B) negatively-charged (120 nm) (n≥6) and (C) non-functionalized NPs (99 nm) (n≥6) fail to induce mucin aggregation since the aggregate size remained below 300 nm throughout 72 hrs incubation. (D) Positively-charged NPs (160 nm) (n≥5) alone can not generate large aggregates in the same Hanks' solution. Smaller size positively-charged (57 nm) (n≥6) promote larger mucin aggregates (E). At the same time, negative controls show that (F) positively-charged NPs (57 nm) (n≥5) or (G) mucin alone (1 mg/L, n≥5) can not generate large aggregates. Various concentrations (solid circles: 100 µg/L, solid triangles: 1 mg/L, solid squares: 10 mg/L) of positively-charged, negatively-charged and non-functionalized NPs (160, 57, 120 and 99 nm) were added to mucin solution (1 mg/L). Significant mucin aggregates were found at 10 mg/L and 1 mg/L of positive NPs. The size of mucin-NPs aggregates were determined with DLS as described previously [37].

To understand the possible mechanisms of how surface charge modifications on NPs affect mucin rheological properties, we examined the effect of different NP surface charges on mucin aggregation and gelation. NPs with non-functionalized, positive, or negative surface charges were added to mucin samples to monitor the mucin gel size change with dynamic laser scattering (DLS) after 0, 1, 3, 5, 24, 48 and 72 hrs. DLS is a well established technique used to measure the hydrodynamic diameter of mucin gel aggregates [37]. In Figure 1A, it is clearly demonstrated that a steady concentration-dependent increase in mucin aggregate size was induced by positively-charged amine (-NH_2_) polystyrene NPs (160 nm) at 1 mg/L and 10 mg/L. Positively-charged NPs (1 mg/L) caused mucin to aggregate from the original 240 nm to 1000 nm within 24 hrs and plateaued throughout 72 hrs. In comparison to the NP-only control (no mucin) at 1 mg/L, only minor aggregation was found throughout the 72 hrs (Figure 1D). On the other hand, 10 mg/L of positively-charged NPs induced a quick rise in mucin aggregation starting from the 1^st^ hour (∼2000 nm) to the 5^th^ hour (∼2900 nm). At the 24^th^ hour, the average mucin gel size increased rapidly to around 5500 nm, while it plateaued around 72 hrs at 6300 nm (Figure 1A). Comparing the 10 mg/L treatment group to the mucin-only control (Figure 1G), there is a 19 and 24 time increase in mucin aggregate size in the corresponding 24 and 72 hrs. However, at much lower concentrations, positively-charged NPs (100 µg/L) failed to induce significant mucin aggregation (Figure 1A). In addition, multiple concentrations of negatively-charged (-COOH based, 120 nm) and non-functionalized (99 nm) NPs yielded a basal size of about 200 nm (Figures 1B & C). The results show that negatively-charged and non-functionalized NPs are not able to associate effectively with mucins (Figures 1B & C). Therefore, our data showed that only positively-charged NPs can effectively promote mucin aggregation. A NP size-dependent effect on mucin aggregation was subsequently investigated. Figure 1E illustrated positively-charged 57 nm NPs generated more pronounced mucin aggregates than 160 nm under the same conditions. The control experiment (Figures 1D & F) indicated that 10 mg/L of 160 and 57 nm positive charged NPs generated only slight self-clumping. Furthermore, 57 nm NP-induced mucin aggregates were approximately 30 times larger than the mucin-only control (Figures 1E & G). The mucin-only control remained at the hydrodynamic diameter of about 200 nm throughout 72 hrs (Figure 1G). These results suggested that NP-induced mucus aggregation occurred mainly as a result of the interactions between NPs and mucin instead of self-aggregation of NPs or mucins.

### SEM images of nanoparticles-induced mucin gel complex

**Figure 2:**
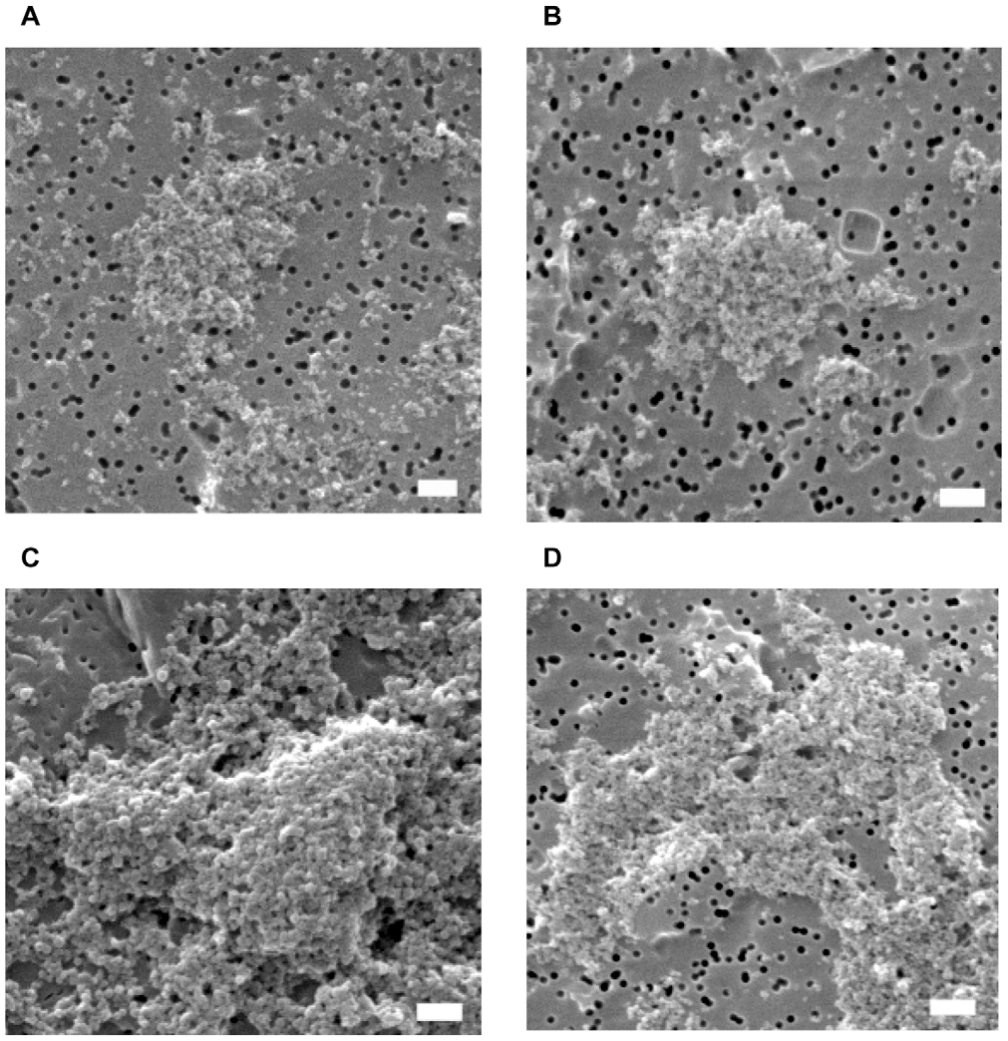
SEM images of mucin-NP complexes. SEM micrographs of mucin-NPs complexes with 160 nm (A, C) and 57 nm (B, D) positively-charged NPs after 5 hrs (A, B) and 72 hrs (C, D). There SEM images clearly demonstrate that NPs and mucins aggregate together forming large mucin-NPs gels. The black holes in the images are the pores in Isopore membranes. The scale bar is 1 µm.

To provide a visual representation that further establishes the relationship between NP concentrations and the size of mucin gels, aggregated mucin size was determined by SEM images. Mucin samples containing 10 mg/L of 57 nm and 160 nm positively-charged NPs were prepared. The resulting mucin gels were collected at 5 hrs and 72 hrs. Mucin gels were filtered through 0.22 µm Isopore membranes, fixed and imaged with SEM. Sizes of mucin aggregates presented in Figures 2A–D confirm the size measurements from DLS. It is also evident that longer incubation resulted in larger NP-mucin aggregates for 57 nm NPs (Figures 2B & D) and 160 nm NPs (Figures 2A & C), which is consistent with DLS measurements (Figures 1A & E). In addition, SEM images demonstrated that these aggregated mucin gels were associated with NPs forming NP-mucin gel complexes.

### Positively-charged nanoparticles serve as mucin gel crosslinkers

**Figure 3:**
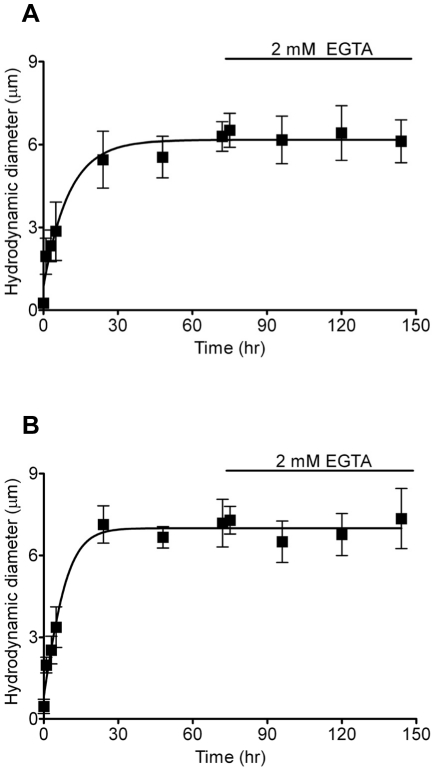
Inability of EGTA (Ca^2+^ chelator) to disperse NPs-induced mucin aggregates. Positively-charged NPs with sizes of 160 nm (A) and 57 nm (B) at 10 mg/L induced mucin (1 mg/L) to aggregate forming large size mucin-NPs aggregates (∼6 µm) in 72 hrs (n≥6). EGTA (2 mM) was added to chelate Ca^2+^ ions that can crosslink mucins forming gels. However, EGTA can not disperse mucin-NPs aggregates after 72 hrs incubation.

Calcium ions have been shown to act as a vital crosslinker in the formation, stability and condensation of mucin gels [22]. Our previous results have shown that gels crosslinked by Ca^2+^ can be dispersed by Ca^2+^ chelators (e.g. EDTA) [34]. To further understand the role that positively-charged NPs play in promoting mucin aggregation and gelation, we tested whether positively-charged NPs can serve as crosslinkers replacing Ca^2+^ ions. Positively-charged NPs (10 mg/L) of 160 nm and 57 nm were added to mucin solution (1 mg/L) and incubated for 72 hrs before applying 2 mM EGTA (Ca^2+^ chelator). The pH was kept in a range of pH 7.4 by Tris-HCl and MES. The changes of aggregated mucin size were subsequently monitored after 3, 24, 48, and 72 hrs. Figures 3A & B demonstrated that calcium chelation by EGTA was unable to significantly reduce aggregated mucin size caused by 160 nm or 50 nm NPs. This result further supports the role of positively-charged NPs as Ca^2+^ crosslinkers.

### Positively-charged nanoparticles retards mucin hydration rate

**Figure 4:**
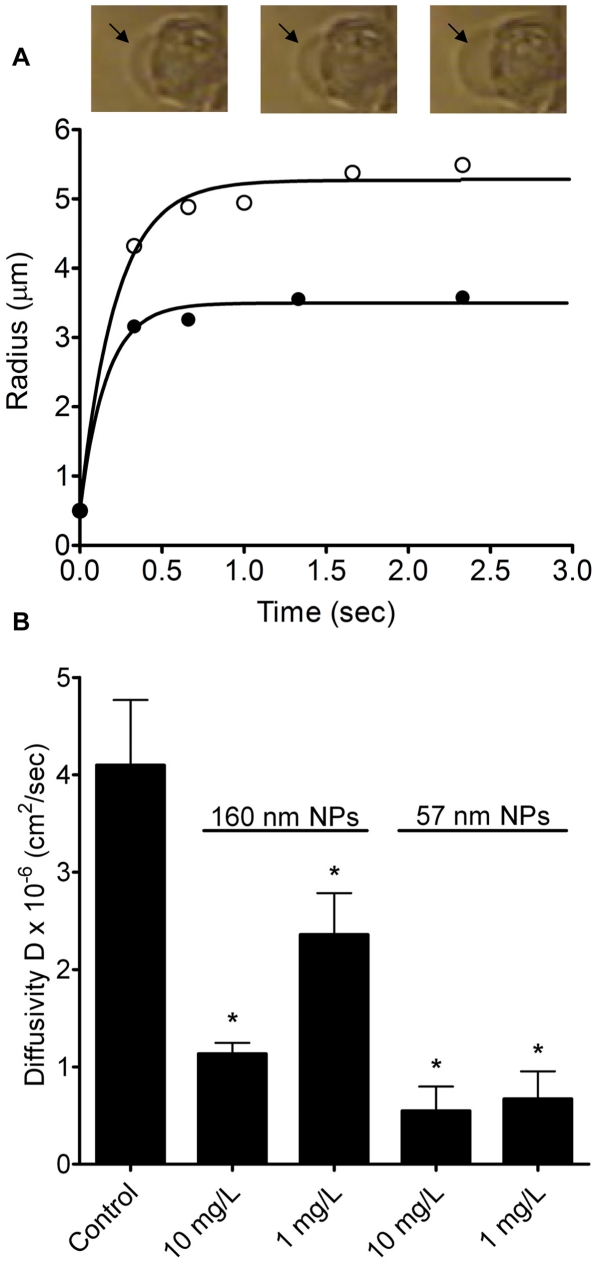
Effects of NPs on swelling kinetics and diffusivity of secretory granule matrix. (A) Data points of swelling kinetics of secretory granule matrices of A549 cells were fit with equation (1). Exocytosis was triggered by ionomycin. Two representative lines are displayed here. The control (NP free; open circles) shows a higher rate of swelling of newly released mucin network than the swelling rate of mucin matrix when exposed to positively-charged NP (160 nm, 1 mg/L, solid circle). The black arrows indicate the swelling of exocytosed mucin matrix at different time points. (B) Positively-charged NPs hinder the rate of mucin diffusivity (hydration). Mucin post-exocytotic swelling from A549 cells was significantly reduced by positively- charged NPs (160 and 57 nm). This protocol to estimate mucin diffusivity of newly released mucin gels from A549 cells was adapted from our previous study [65]. Data are shown as mean±SD (n≥10). NP treated groups are significantly different from the untreated control at p<0.005 as indicated by *.

Knowing that positively-charged NPs can disturb mucin rheological properties, we then tested whether positively-charged NPs could impair mucin hydration by utilizing an *in vitro* mucin swelling kinetics functional assay (see methods). A representative graph comparing the swelling kinetics of newly exocytosed mucin networks, between the control and positively-charged NP treatment, is shown in Figure 4A. The evidence indicated that positively-charged NPs impeded the swelling rate (∼hydration) of mucin networks (Figure 4A). Converting swelling rate into diffusivity (D) using equation (2) yielded similar results. The measurements showed that positively-charged NPs (10 mg/L) of 160 and 57 nm retarded the rates of mucin diffusivity approximately 3.6 and 7.5 times, while 1 mg/L of 160 and 57 nm decreased mucin diffusivity about 1.7 and 6.1 times, correspondingly, when compared to the control (NP free) (Figure 4B). Within the same category of NP size, there is also a concentration-dependent effect on reducing the rate of mucin diffusivity. Higher NP concentration (10 mg/L) yields a slower rate of mucin diffusivity than lower NP concentration (1 mg/L). Our data indicates that positively-charged NPs can reduce the rates of mucin hydration and diffusivity, possibly leading to an elevated mucus viscosity and dysfunctional mucociliary transport.

## Discussion

Mucus is mainly composed of large and heavily glycosylated glycoproteins called mucin. The gel-forming mucins rapidly hydrate after exocytosis and due to their tangle network properties, anneal with other mucins to form a protective barrier at the airway-surface liquid layer. The mucin gel layer lines the epithelial surface of various organs such as the vaginal tract, eyes, gastric wall and pulmonary lumen [38]. Mucus in the airway of lungs serves as an innate immune defense against inhaled particulates, bacteria and viruses [39]. Maintenance of the airway protection mechanism stems from the delicate balance between normal mucus production, transport and clearance. The mucin polymer network of mucus has a characteristic tangled topology [40]. Since the rheological properties of mucus are governed mainly by the tangle density of mucin polymers, which decreases with the square of the volume of the mucin matrix, the mucin network hydration (degree of swelling) is the most critical factor in determining the rheological properties of mucus [22]. The diffusivity of mucin matrices, which is closely related to mucin viscosity [22], [36], can be calculated from polymer swelling kinetics. Based on the polymer network theory, polymer diffusivity is inversely proportional to its viscosity [41], [42], [43]. Thus, lower rate of mucin diffusivity is associated with higher viscosity, less dispersed and less transportable mucins that appear to characterize the clinical symptoms of thick mucus accumulation and obstruction commonly found in asthma, COPD and CF [29], [35], [44]. Although chronic exposure to NPs has been shown to induce excessive mucin synthesis [45] and increase the risk of COPD [30], the connection between NPs and poorly dispersed viscous mucus has not been explored.

Due to the versatile application of NPs in nanocarrier delivery systems and commercial products, NPs with varying surface functionalization and characteristics are widely utilized [9], [46]. The polyanionic nature of mucin glycoproteins allows for possible electrostatic interactions with oppositely charged NPs. In this study, we investigated how surface charges and size modifications of NPs influence mucus rheological properties. Mucus hydration can be significantly impeded by a change in electrolyte concentration in airway-surface liquid (ASL) [22]. Calcium ion concentration has been shown to increase from 1 mM to 4 mM in the ASL of CF patients, hindering proper mucus swelling and hydration [47] and leading to respiratory health problems. So far there is no evidence documenting ASL abnormalities in NP-induced airway diseases. However, the mechanism by which NPs can cause changes in the rheological properties of mucus is still unknown.

Gastric mucin was used as a model mucin in our investigation as it shares many similarities with airway mucins [48], [49], [50]. Our approach is consistent with previous studies that used gastric mucin to examine the viscoelastic properties and biological applications of mucins [51], [52], [53], [54], [55], [56], since there is currently no commercial mucin sample available that can be preserved in the native state. We utilized DLS to probe the interactions between functionalized NPs and mucin. DLS is a common technique implemented to measure the Brownian motion of mucin polymer in solvent [21], [22]. One of the major advantages of this technique is that it enables mucin structure and dynamics to be examined in the native state without chemical fixation, which minimizes protein denaturation and artifacts [21], [22]. The diffusion constant that was calculated from Brownian motion measurement is inversely proportional to the hydrodynamic diameter of the measured particle; low diffusion constant usually correlates with large mucin gelation/aggregation [21], [22]. As demonstrated by Figures 1A, E & 2A–D, positively-charged NPs can promote mucin aggregation (NP-mucin gel complexes) in a concentration- and size-dependent manner. The mechanism may involve electrostatic attraction between positively-charged NPs and the polyanionic sites on mucin such as the sialic, sulfate and carboxyl functional groups promoting mucin aggregation. By the same notion, mucin polyanionic charges are neutralized by cationic calcium counterions [22] which condenses the mucin matrix and facilitates gel formation [57], [58]. On the other hand, negatively-charged and non-functionalized NPs failed to generate significant mucin aggregation. Other studies have shown that hydrophobic and mucin-mucin interactions play vital roles in pH-induced gelation of gastric mucin [59]. However, the hydrophobic moieties on non-functionalized NPs failed to promote significant mucin gelation in our study. This inability might be partially due to the low mucin concentration (1 mg/L) and the neutral pH (buffered at 7.4). The negatively-charged NPs are likely to repel anionic sialic, sulfate and carboxyl functional groups on mucin thereby hindering mucin aggregation [21], [22]. Another possible mechanism could be that these negatively-charged NPs can chelate divalent ions (e.g. Ca^2+^) in the solution, which play a critical role in mucin gel crosslinking [22].

In addition, our data validated that smaller NPs can induce the formation of larger size mucin aggregated gels more effectively (Figures 1E, 2B & D). The resulting larger mucin gels can possibly be elucidated by differential charge densities on NPs [60]. Our findings are supported by other studies showing that positively-charged chitosan coated NPs, or amine-modified NPs, were highly adhesive to polyanionic mucus gels [61], [62]. Viral particles with highly dense positive charges have also been proposed to be more mucoadhesive [38].

After establishing that positively-charged NPs can promote mucin gelation, our data suggests that positively-charged NPs may indeed act as network crosslinkers. Figures 3A & B confirmed that EGTA chelation of indigenous mucin network crosslinker Ca^2+^ ions was unable to disperse the NP-induced mucin aggregation/gelation. Therefore, positively-charged NPs crosslink the polyanionic tangle networks via electrostatic interactions. This evidence further validates the idea that positively-charged NPs effectively replace the natural role of Ca^2+^ ions and highlights the health danger with irreversibly strengthening the crosslinking within mucin gels.

To demonstrate the harmful consequence that positively-charged NPs can have through their crosslinking ability on mucus rheological properties, a swelling kinetics functional assay was used. We determined the direct effect of positively-charged NPs on the mucus hydration rate by measuring the swelling kinetics of newly exocytosed mucin matrices from human lung A549 cells under positively-charged NP exposure. A549 cells are a representative *in vitro* model system for studying mucin swelling kinetics as they express both major respiratory MUC 5AC and MUC 5B mucin proteins [63], [64]. Our study showed that positively-charged NPs hindered the rates of mucin matrix hydration and diffusivity in both a concentration- and size-dependent manner (Figures 4A & B). In accordance with the polymer network theory (lower diffusivity correlates with higher viscosity) [41], [42], [43], our data indicated that positively-charged NPs decrease the rate of mucin diffusivity and can increase the viscosity of mucin network; the effect of which is further enhanced by smaller sized NPs. Our experimental data has provided the first mechanistic link between positively-charged NPs and altered rheological properties of mucus.

### Conclusions

The positively-charged NPs can crosslink mucins, thus forming NP-mucin gel complexes. The effects of which can potentially induce the formation of viscous mucus and hinder proper mucus hydration and dispersion, leading to impaired mucociliary transport in various epithelial mucosa. The outcome of viscous mucus accumulation could exacerbate pulmonary symptoms of diseased individuals and potentially elevate chances of morbidity in healthy pulmonary systems [23]. As in the case of COPD, CF, or asthma patients, positively-charged NPs could worsen the problem by further thickening the mucus. The complications may include additional reduction in the airway flow or the promotion of chronic bacterial infection. This report also indicates some possible undesirable side effects of drug delivery using positively-charged nanocarriers via epithelial mucosa. These NPs might lead to drug entrapment within mucus, impedance of proper mucus dispersion, and transportability. Our study also found that non-functionalized and negatively-charged NPs have less impact on the rheological properties of mucus. This finding provides the necessary knowledge needed for the safety consideration when using positively-charged functionalized NPs.
